# A novelty in *Ceratozamia* (Zamiaceae, Cycadales) from the Sierra Madre del Sur, Mexico: biogeographic and morphological patterns, DNA barcoding and phenology

**DOI:** 10.3897/phytokeys.156.53502

**Published:** 2020-08-21

**Authors:** Lilí Martínez-Domínguez, Fernando Nicolalde-Morejón, Francisco G. Lorea-Hernández, Francisco Vergara-Silva, Dennis Wm. Stevenson

**Affiliations:** 1 Posgrado en Ciencias Biológicas, Instituto de Biología, Universidad Nacional Autónoma de México, 3er. Circuito Exterior, Ciudad Universitaria, 04510, Coyoacán, CDMX, Mexico; 2 Laboratorio de Taxonomía Integrativa, Instituto de Investigaciones Biológicas, Universidad Veracruzana, Xalapa, 91190, Veracruz. Mexico; 3 Red de Biodiversidad y Sistemática, Instituto de Ecología, A.C., Xalapa, 91073, Veracruz. Mexico; 4 Laboratorio de Sistemática Molecular (Jardín Botánico), Instituto de Biología, Universidad Nacional Autónoma de México, 3er. Circuito Exterior, Ciudad Universitaria, 04510, Coyoacán, CDMX. Mexico; 5 The New York Botanical Garden, Bronx, New York, 10458-5120, USA

**Keywords:** cloud forest, cycads, Guerrero, integrative taxonomy

## Abstract

*Ceratozamia* is a genus of cycads occurring in eastern Mexico and Central America. In this study, we describe a new species from the Pacific region of Mexico in Guerrero state. This locality represents the most northwestern Mexico distribution for the genus. We focus the comparison of this species with the most geographically proximate and phenotypically relevant lineages for this taxon. We followed an integrative taxonomy approach to evaluate the classification of these species, including geographic location, morphology, DNA barcoding and phenology as primary sources of systematic data. Within the morphological dataset, reproductive structures are described in detail and new characters are proposed for microsporophylls. The comparative morphology of these structures facilitated the elucidation of differences in forms and species for identification. The two chosen DNA barcoding markers – namely, the chloroplast genome coding region *matK* and the nuclear ribosomal internal transcribed spacer (ITS) region – had low divergence, allowing only 61% of species identification, suggesting slow molecular evolutionary rates. Besides employing these three basic sources of evidence, we introduced phenology as additional information for species circumscription. In addition, this work includes a brief review of the genus at the species-level. This is therefore the most recent review for *Ceratozamia* across its full geographic range (latitudinal and elevational). Overall, this work further contributes to a comprehensive framework for systematic studies in Mexican cycads.

## Introduction

The Sierra Madre del Sur (SMS) is a biogeographic province assigned to the Mexican Transition Zone, which includes neartic and neotropical ecosystems (Marshall and Liebherr 2000). This Mexican province runs northwest-southwest parallel to the Pacific Ocean coast throughout 1,200 km. It has great diversity of species, due to its high climatic and topographical heterogeneity (Morrone 2005; Santiago-Alvarado et al. 2016). Its rugged orography has been influenced by ancient geological events. In parallel, regional climatic cycles are relevant to explain its ecological heterogeneity, including overlaps between the Neotropical and Nearctic biotas, both of which house high species richness and endemics (Morrone 2010). The SMS has been divided into three subprovinces, in which the eastern Sierra Madre del Sur subprovince is the most heterogeneous in structural composition. This subprovince includes two districts: the Guerrero and Oaxaca highlands (Morrone 2017).

One of the least represented habitats in the SMS, and perhaps the least studied, is the SMS cloud forest. This type of vegetation is distributed unequally between 600 and 1,800 meters of elevation, and has a very disjointed and fragmented range caused by different climatic cycles (Luna-Vega et al. 1999). Moreover, the cloud forest is the most diverse habitat in Mexico per unit area and is found in small fragmented zones mainly in the Guerrero and Oaxaca highland districts (Rzedowski 1996; Challenger 1998).

The distribution of *Ceratozamia* Brongn. (Cycadales) is restricted to areas with high humidity in the main mountain systems of Mexico and Central America. The genus occurs in a spectrum of habitats: evergreen tropical forest, oak-pine forest, and cloud forest in the Sierra Madre Oriental (SMO), Sierra Madre de Chiapas (SMCh), SMS proper, and mountains of eastern Central America. The greatest diversity of this genus is found in the SMO. In the SMS, *Ceratozamia* has only been reported in Oaxaca (Vovides et al. 2004; Contreras-Medina 2016), but the north central portion of the SMS also may have suitable habitats for the genus. This is particularly so in Guerrero, which has small patches of cloud forest and is part of this biogeographic province (Luna-Vega et al. 1999).

*Ceratozamia* is easily diagnosed by the presence of two horns at the distal end of the sporophylls (Norstog and Nicholls 1997). This genus is one of the most complex morphological assemblages within the family Zamiaceae, because some of its species show considerable intrapopulation variation and most characters commonly used to diagnose species in other cycad genera are polymorphic (Martínez-Domínguez et al. 2017). Taxonomic and molecular systematic studies have contributed to clarify species identification and to provide taxonomic revisions with keys at the regional level (e.g. Pérez-Farrera et al. 2009; Martínez-Domínguez et al. 2016, 2017, 2018a). Generally, quantitative morphological characters provide limited information among closely related species (Martínez-Domínguez et al. 2017). However, reproductive characters have been poorly studied at the species level, both morphologically and in terms of phenological cycles. Detailed studies and descriptions of organs in the reproductive structures – mainly micro- and megasporophylls – could yield potentially useful diagnostic characters in the genus (Martínez-Domínguez et al. 2018a, b).

Recently, during a review of collections of *Ceratozamia* deposited at the FCME herbarium, our research group found a specimen collected in 1984 from the state of Guerrero. However, this material lacked reproductive structures indispensable for unequivocal identification. Because the previously known biogeographic pattern of this genus was restricted to southeastern and central Mexico, this discovery was a novelty, as the corresponding coordinates would represent the northernmost locality of *Ceratozamia* for the Mexican Pacific. Given that similar specimens from the same geographic point had not been collected again, we explored the corresponding area in search of this underdescribed population. After conducting extensive fieldwork in Guerrero to collect fresh material and monitor the attendant reproductive process, we compared the new specimens with all known species in the genus with an initial focus on similar species using geographic and morphological criteria. Formally, we adopted an integrative taxonomic approach (*sensu* De Salle et al. 2005; Goldstein and DeSalle 2010) that involves a set of inferential rules for corroboration or refutation of species hypotheses based on multiple sources of evidence (‘taxonomic circle’ *sensu*DeSalle et al. 2005). Elsewhere, we have successfully used this approach for species-level identification in *Ceratozamia*, explicitly considering intraspecific morphological variation (Martínez-Domínguez et al. 2017). To identify this unidentified herbarium sheet and additional specimens from two Guerrero populations, which ultimately turned out to match the new taxon herein described, we use geographic location information, both qualitative and quantitative morphological characters, character-based DNA barcoding and phenological data. Finally, we have explored how this taxonomic discovery might alter our understanding of biogeographic and evolutionary patterns in *Ceratozamia*.

## Materials and methods

### Specimen collection and morphological character coding

Twenty-one specimens were collected for the newly described taxon from two localities in Guerrero, Mexico. Leaf tissue was collected from all individuals for DNA sequencing and preserved in silica-gel. In total, we sampled 8 to 10 specimens approximately per population for *Ceratozamia robusta* Miq., and *C. subroseophylla* Mart.-Domínguez & Nic.-Mor., three and two populations, respectively (Appendix I). These specimens were collected during 2014 and 2017 by our research group, which have been deposited at the CIB and MEXU. In these collections, we have included the type locality and associated populations that correspond to the distribution range. Species selection was further based upon morphological similarities with the initial *Ceratozamia* specimen from Guerrero. For this species, a set of 40 continuous, quantitative characters were coded along with another series of 39 discrete, qualitative characters. Additionally, we examined all herbarium specimens for the 30 species recognized in the genus, which are deposited at CIB, ENCB, FCME, MEXU, US, NY, and XAL. From the herbarium specimens for all species, we evaluated a set of 25 continuous and 23 discrete characters.

### Biogeographic information for specimens

Herbarium specimens of *Ceratozamia* were reviewed and their geographic coordinates were used to a compile a database. This information was verified in the geographic information system ArcMap GIS v.10.2. Ambiguous and/or doubtful geographic data were omitted; in cases where precise locality data were available, we georeferenced each locality with Google Earth Pro (2020) (http://earth.google.es/). To determine elevation for all registered samples, points of occurrence were superimposed on the ‘digital elevation model’ available from CONABIO (Guevara and Arroyo-Cruz 2016). The vegetation type for the populations of Guerrero taxon was characterized and classified following the biogeographical provinces of cloud forest according to CONABIO (2008).

### DNA extraction, PCR amplification and DNA sequencing

Genomic DNA was extracted from five individuals for each population collected in Guerrero using the DNeasy Plant Mini Kit (QIAGEN, Hilden, Germany). Additionally, DNA was extracted from a single individual for *Ceratozamia chamberlainii* Mart.-Domínguez, Nic.-Mor. & D.W. Stev., *C. delucana* Vázq.Torres, A. Moretti & Carvajal-Hern., *C. mexicana* Brongn., and *C. totonacorum* Mart.-Domínguez & Nic.-Mor. Samples from these taxa were not included in previous works (cf. Nicolalde-Morejón et al. 2011; Martínez-Domínguez et al. 2017; Medina-Villarreal et al. 2019). For amplification of the nuclear ribosomal internal transcribed spacer (ITS) region, primers 5a fwd (5’CCTTATCATTTAGAGGAAGGAG3’) and 4 rev (5’TCCTCCGCTTATTGATATGC3’) were used, whereas primers fwd (5’ATACCCCATTTTATTCATCC3’) and rev (5’GTACTTTTATGTTTACGAGC3’) were used for the maturase K (*matK*) chloroplast genome locus. The latter region was amplified in *Ceratozamia subroseophylla* (an ITS region sequence was previously available) and included in the molecular matrix. Choice of markers followed previous publications where they performed best in terms of number of diagnostic sites (Martínez-Domínguez et al. 2016, 2017). Amplification products were visualized in 1% ethidium bromide-stained, agarose gels. Bands of the expected molecular weight were purified with a QIAquick PCR Purification Kit (QIAGEN) and sent to Laboratorio de Secuenciación Genómica de la Biodiversidad y la Salud (LANABIO; Instituto de Biología, UNAM) for automated DNA sequencing. All DNA sequence data were deposited in GenBank.

### Character-based DNA barcoding

Electropherograms were edited and assembled using the program Sequencher v.4.8 (Gene Codes Corp., Ann Arbor, MI, USA). Sequences were aligned in BioEdit v.7.0.9 using the ‘multiple alignment’ option in Clustal X (Thompson et al. 1997; Hall 1999). We manually checked and edited these alignments with MacClade v.4.03 (Maddison and Maddison 2001). A matrix of these sequences, plus a subset of sequences for *Ceratozamia* including previously published ITS and *matK* sequences currently available in GenBank, was then saved in Nexus format for further use (Suppl. material 1: File S1). All markers were assembled with SequenceMatrix v.1.7.8 (Vaidya et al. 2011). The resulting dataset corresponding to all species currently and correctly ascribed to the genus in Mexico (Nicolalde-Morejón et al. 2011; Martínez-Domínguez et al. 2017) was subject to character-based DNA barcoding in CAOS (Character Attribute Organization System; Sarkar et al. 2008). Following the recommendations in the CAOS manual, a guide tree was prepared using the molecular matrix of *Ceratozamia* DNA sequences. The total length of the concatenated ITS and *matK* data matrix was 1813 nucleotide sites, including gaps. Cladistic analyses on this matrix in TNT v.1.5 (Goloboff et al. 2008) generated nine shortest, equally parsimonious trees. Because relationships in the corresponding strict consensus tree were not completely resolved (Suppl. material 2: Fig. S1), this tree was manually edited to avoid polytomies, preserving the arrangement of resolved clades in all trees. The topology of the resulting guide tree was saved in the Nexus format for further CAOS analyses. Identification of DNA diagnostics was carried out with the *P-gnome* program. Only simple pure (‘sPu’ *sensu*Sarkar et al. 2008) characters (i.e. attributes) with confidence value of 1.00 were selected.

### Reproductive phenology data

The populations registered for the Guerrero taxon, as well as two populations for both *Ceratozamia robusta* and *C. subroseophylla*, were monitored for phenological observations of ovulate strobili (Appendix I). In addition, herbarium specimens of known wild collections in CIB, MEXU, and XAL were examined for complementary information in this regard (Suppl. material 3: File S2). All observations were categorized into one of the following three states, which were determined according to Martínez-Domínguez et al. (2018b): (i) receptivity (R); (ii) late ovulate (LO); and (iii) degraded (D). Determining phenological patterns for other species in the genus was not possible due to the lack of reproductive structures in herbaria.

## Results

### Biogeographic patterns

**Figure 1.**
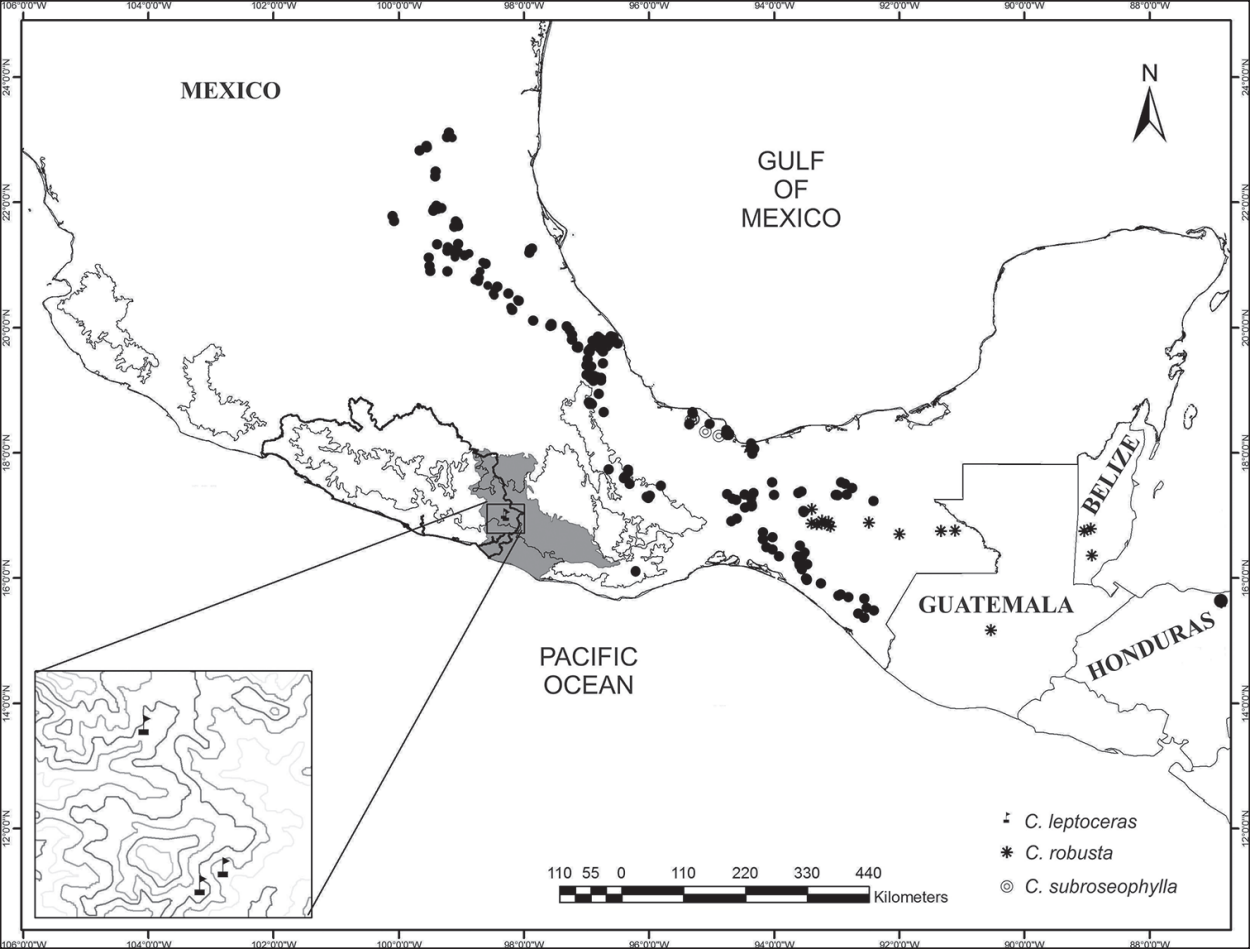
Distribution map of the genus *Ceratozamia* (black solid circles). Distributions for species morphologically similar to *Ceratozamia leptoceras* are represented with an asterisk and a double circle. Guerrero state and Putla subregion of cloud forest are shown with outline and shaded area, respectively. Inset: points corresponding to the Guerrero mountain range where *Ceratozamia* was collected.

**Figure 2.**
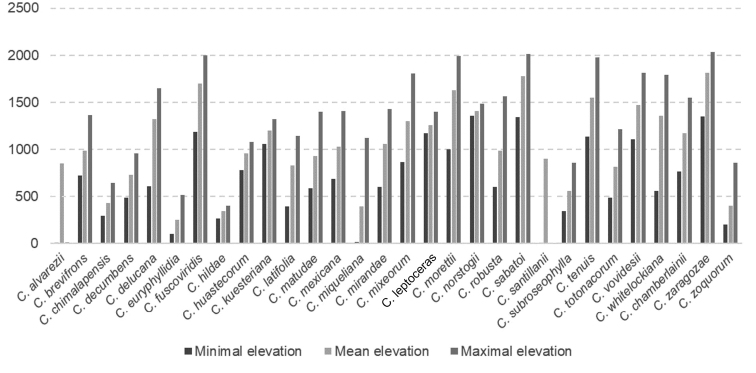
Elevational distribution of *Ceratozamia*. Intervals of altitudinal distribution of *Ceratozamia* species are shown.

The genus *Ceratozamia* has a continuous but restricted distribution along two of the major mountain ranges in Mexico – namely, the Sierra Madre Oriental (SMO) and the Sierra Madre del Sur (SMS), Belize, and some lowlands in Los Tuxtlas (Veracruz, Mexico) and Honduras (Fig. 1). Elevational ranges for most species are broad, usually occurring between 800 to 1,400 meters. However, some species are distributed at the extremes of this general pattern. *Ceratozamia miqueliana* H.Wendl., can occur at elevations as low as 19 meters, and *C. zaragozae* Medellín-Leal at elevations up to 2,030 meters (Fig. 2). The species with the greatest elevation range are *C. fuscoviridis* W. Bull, *C. miqueliana*, *C. robusta*, and *C. zoquorum* Pérez-Farr., Vovides & Iglesias. In turn, the two species with the narrowest range of elevational variation are *C. hildae* G.P.Landry & M.C.Wilson and *C. euryphyllidia* Vázq.Torres, Sabato & D.W.Stev. The new populations registered from Guerrero are the northernmost for the genus on the western ranges of the coastal Pacific Ocean region; this location corresponds to the northern end of the SMS province, from 1,100 to 1,400 meters of elevation (Fig. 1).

The *Ceratozamia* taxon herein described from Guerrero is found in relictual cloud forest and the transition zone between cloud forest and oak forest on rocky limestone slopes. In contrast, *C. robusta* is found in evergreen tropical forest and oak forest, whereas *C. subroseophylla* only inhabits evergreen tropical forest. These two related *Ceratozamia* species occur on clay soils with isolated rocks of volcanic origin, whereas the Guerrero taxon occurs on karstic rocks. In the context of classifications of biogeographical provinces of cloud forest, the Guerrero taxon occurs in southern coastal mountain range and the Putla subregion.

### Comparisons of quantitative and qualitative morphological characters

In terms of vegetative morphology, three general groups of species within *Ceratozamia* can be distinguished. These groups include plants with (i) very wide leaflets, between (2.5) 4.5–17.6 cm, oblong to oblanceolate and obovate, (ii) wide leaflets of 2.3–4.6 cm, lanceolate to linear, and (iii) narrow leaflets, 0.8–2.2 cm wide, linear to lanceolate. The first group includes the species *C. miqueliana*, *C. zoquorum*, *C. latifolia* Miq., *C. huastecorum* Avendaño, Vovides & Cast.-Campos, *C. euryphyllidia*, *C. decumbens* Vovides, Avendaño, Pérez-Farr. & Gonz.-Astorga, *C. hildae*, *C. morettii* Vázq.Torres & Vovides, *C. santillanii* Pérez-Farr. & Vovides, *C. hondurensis* J.L.Haynes, Whitelock, Schutzman & R.S.Adams, *C. chamberlainii*, and *C. totonacorum*. Plants belonging to this group of species also have hypogeous stems, few leaves, and small ovulate strobili, with the exception of *C. miqueliana*, *C. chamberlainii*, and *C. totonacorum*, which have ovulate strobili up to 30 cm long and epigeous stems. The second group has epigeous stems, many leaves, and cylindrical and long ovulate strobili; this group contains *C. mexicana*, *C. subroseophylla*, *C. robusta*, *C. whitelockiana* Chemnick & T.J.Greg., *C. mixeorum* Chemnick, T.J.Greg. & Salas-Mor., *C. delucana*, and *C. brevifrons* Miq. The third group has epigeous and semi-epigeous stems and greater variation in relation to the size of ovulate strobili; it includes *C. zaragozae*, *C. norstogii* D.W.Stev., *C. alvarezii* Pérez-Farr. Vovides & Iglesias, *C. mirandae* Vovides, Pérez-Farr. & Iglesias, *C. vovidesii* Pérez-Farr. & Iglesias, *C. matudae* Lundell, *C. tenuis* (Dyer) D. W. Stev. & Vovides, *C. sabatoi* Vovides, Vázq.Torres, Schutzman & Iglesias, *C. chimalapensis* Pérez-Farr. & Vovides, *C. fuscoviridis*, and *C. kuesteriana* Regel. In preparation for the integrative taxonomy analysis, morphological comparisons revealed that the taxon from Guerrero has epigeous stems, median leaflets of 1.9–2.8 cm width, and long ovulate strobili. These data strongly suggest affinity of the Guerrero taxon to the second group.

**Figure 3.**
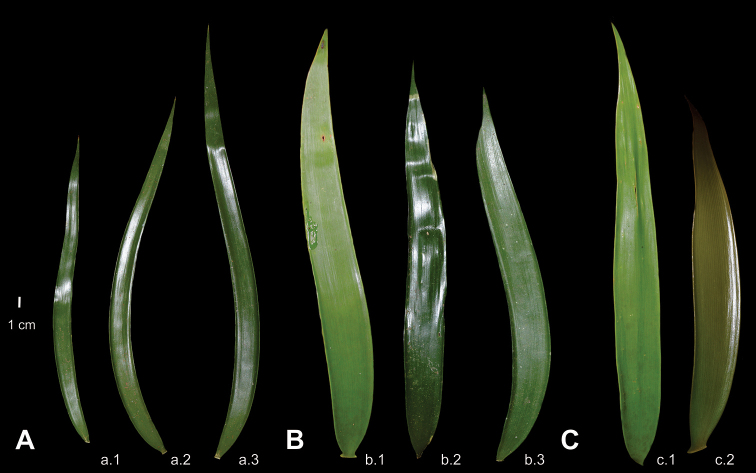
Leaflet variation at the population level **A***Ceratozamia leptoceras*, a.1, a.2 San Pedro Cuitlapan, a.3 Riverbank “Chipili” **B***C. robusta*, b.1 Cañón del Sumidero, b.2, b.3 Cuchumbak **C***C. subroseophylla*, c.1 Sinapan, c.2 “El Vigía”. All leaflets were collected from middle and right side of leaf with exception of two first leaflets for *C. leptoceras* (left).

In this context, the Guerrero taxon has a close morphological similarity to *Ceratozamia subroseophylla* and *C. robusta*; however, it has differences when compared to all species within the second group (see the taxonomic key below, for more details). Further morphological comparisons between these three entities reveal that only a few vegetative morphological characters provide support for species delimitation. The detailed morphological differences between *C. subroseophylla*, *C. robusta* and the Guerrero taxon are listed in Tables 1, 2. In this regard, the leaves of the Guerrero taxon show great morphometric affinity with *C. robusta* and *C. subroseophylla* with a leaf length of ranging from 1.20 to 2.80 meters. In contrast, the leaf position of the new taxon is descending, whereas in the other two species it is erect (Table 1). Additionally, the Guerrero taxon bears linear and membranaceous leaflets in contrast to *C. robusta* and *C. subroseophylla*, which have lanceolate and papyraceous to subcoriaeous leaflets (Fig. 3). Furthermore, the shape of prickles on the petiole are robust in *C. subroseophylla* and *C. robusta*, whereas in the Guerrero taxon the prickles are thin.

**Table 1.** Comparison of diagnostic qualitative morphological characters between *Ceratozamia leptoceras* and morphologically similar species.

Characters	Species		
	*C. leptoceras*	*C. robusta*	*C. subroseophylla*
Leaf color at emergence	*Green with copperish-green petiole*	Dark brown	Yellowish-brown
Leaf position	*Descending*	Ascending	Ascending
Prickles on petiole	*Thin*	Robust	Robust
Leaflet shape	*Linear*	Lanceolate	Lanceolate
Leaflet texture	*Membranaceous*	Papyraceous	Papyraceous
Leaflet base color	Green (brown only in articulation)	Green (yellow in juvenile leaves)	Green (brown in juvenile leaves)
Ovulate strobilus color	*Copperish-green with greyish-black pubescence*	Dark green with dark trichomes	Rosaceous-green with brown trichomes
Ovulate strobilus apex	*Acute*	Acuminate	Mucronate
Megasporophyll horns shape	Straight	Straight	Straight
Megasporophyll distal face form	Prominent	Prominent	Prominent
Microsporophylls horns direction	Straight	Recurved	Recurved
Microsporophylls horns shape	*Thin*	Robust	Robust
Infertile portion of microsporophylls	*Linear*	Rounded	Rounded
Fertile portion of microsporophylls	Deeply lobate	Deeply lobate	Lobate

Autapomorphic character states are shown in italics.

**Figure 4.**
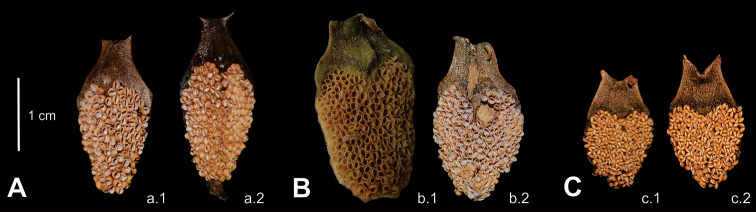
Abaxial view of microsporophylls **A***Ceratozamia leptoceras*, a.1, a.2 San Pedro Cuitlapan **B***C. robusta*, b.1 Cuchumback, b.2 Cañón del Sumidero **C***C. subroseophylla*, c.1 “El Vigía”, c.2 Sinapan. For more detail of differences in character states see Table 1.

The primary diagnostic morphological characters lie in the reproductive structures. However, quantitative characters traditionally used for species distinction overlap in this case, both in length and diameter of ovulate strobili and peduncle length of ovulate strobili (Table 2). The most relevant and diagnostic characters are, instead, the number of megasporophylls per orthostichy and the length of horns in the megasporophylls (Table 2). Moreover, the pollen strobilus in the Guerrero taxon has several differences when compared to species in the second group, especially in terms of microsporophyll morphology: in the Guerrero taxon, the horns of microsporophylls are straight, thin, the infertile portion is linear, and the fertile portion is deeply lobate (Fig. 4).

**Table 2.** Comparison of diagnostic quantitative morphological characters between *Ceratozamia leptoceras* and morphologically related species; values are given in centimeters. The reproductive structures were measurements at maturity.

Characters	Species		
	*C. leptoceras*	*C. robusta*	*C. subroseophylla*
Pairs of leaflets*	22–61	13–58	15–40
Distance between median leaflets	1.8–2.8	2–3.9	0.9–3.9
Length of median leaflets	28–43.5	30.5–44.5	23.5–44.5
Width of median leaflets	1.9–2.8	3.1–3.9	2.4–4
Length of ovulate strobilus	23.5–28	26–40	15.5–23.5
Diameter of ovulate strobili	9.5–11	11.5–14.5	7–10
Length of ovulate strobili peduncle	11–16	5–6.2	9.8–17.5
Number of orthostichies*	8–9	9–12	9–11
Number of megasporophylls per column*	*7–9*	17–20	11–13
Length of megasporophyll horns	*0.60–0.81*	0.38–0.50	0.41–0.62
Length of pollen strobili	*42–45*	60–70	15–30
Diameter of pollen strobili	6.0–7.8	7–8.5	6.2–8
Length of pollen strobili peduncle	13–19	14–17	10–15
Width of microsporophylls	1.09–1.35	1.14–1.80	1.01–1.24
Length of microsporophylls	2.21–2.55	2.33–3.0	1.47–2.80
Length infertile portion of microsporophylls	*0.83–0.96*	0.45–0.65	0.49–0.59
Distance between microsporophyll horns	0.44–0.56	0.55–0.75	0.55–0.42
Length of microsporophyll horns	*0.1–0.23*	0.26–0.40	0.27–0.38

Diagnostic character states are in italics. *Meristic characters.

### Character-based DNA barcoding

Sequences of *matK* and ITS allowed the molecular identification of 19 out of 31 *Ceratozamia* species (Table 3). The ITS region alone granted 45% identification success of recognized species, which increased to 61% after the inclusion of *matK*. Surprisingly, molecular diagnosability determined through character-based DNA barcoding in CAOS indicated that the taxon from Guerrero has two autapomorphies within *matK* [i.e. ‘simple pure’ (sPu) characteristic attributes *sensu*Sarkar et al. 2008] in all five sequenced individuals (G: 1592; C: 1807). No interindividual or intrapopulation polymorphic sites were detected.

Among the species with morphological affinity to the Guerrero taxon, *Ceratozamia robusta* and *C. subroseophylla* were diagnosable with ITS and ITS+*matK*, respectively. Notably, *C. subroseophylla* has a 15 nucleotide deletion in region 569–583 of the aligned matrix; this type of deletion is not present in any other *Ceratozamia* species (Table 3; Suppl. material 4: Table S1) and, thus, represents an autapomorphy. Our samples from Guerrero are more distant genetically from other phenotypically similar species such as *C. mexicana*, *C. mixeorum*, *C. delucana*, *C. whitelockiana* and *C. brevifrons*. The latter three species did not have any diagnostic sites; however, a closer visual inspection of groups generated by CAOS showed divergences with the taxon from Guerrero and greater similarities with *C. miqueliana* and *C. morettii*.

**Table 3.** Species identification using the candidate combination of loci for character-based DNA barcoding in *Ceratozamia*. The dash indicates absence of diagnostic sites.

**Species**	**ITS region**	***matK***	**Total DNA diagnostic sites**
***C. alvarezii***	1	–	1
*C. brevifrons*	–	–	–
***C. chamberlainii***	–	1	1
*C. chimalapensis*	–	–	–
*C. decumbens*	–	–	–
*C. delucana*	–	–	–
***C. euryphyllidia***	1		1
*C. fuscoviridis*	–	–	–
*C. hildae*	–	–	–
C. hondurensis*	–	–	–
*C. huastecorum*	–	–	–
***C. kuesteriana***	6	3	9
***C. latifolia***	1	–	1
***C. leptoceras***	–	2	2
***C. matudae***	4	2	6
C. mexicana*	1	–	1
*C. miqueliana*	–	–	–
***C. mirandae***	–	2	2
***C. mixeorum***	3	–	3
***C. morettii***	–	3	3
*C. norstogii*	–	–	–
***C. robusta***	1	–	1
***C. sabatoi***	1	1	2
***C. santillanii***	2	–	2
***C. subroseophylla***	1	1	2
***C. tenuis***	2	–	2
*C. totonacorum*	–	–	–
***C. vovidesii***	–	3	3
*C. whitelockiana*	–	–	–
***C. zaragozae***	1	–	1
***C. zoquorum***	8	–	8

Gray squares indicate the presence of at least one DNA diagnostic site for the corresponding locus. For species with an asterisk, only ITS sequences are available.

*Ceratozamia kuesteriana* had the greatest number of diagnostic sites (nine in total). This species was followed by *C. matudae* and *C. zoquorum*, with six and five diagnostic characters, respectively. The remaining species of the genus had low values of DNA diagnostics; the number of diagnostic sites by species ranges from one to three DNA diagnostics, and for nine species diagnostic sites were consistently absent (Table 3). In the case of *C. chamberlainii* only a gap in *matK* was recovered as a potential diagnostic site and it was included as a diagnostic character due to its location in a coding region.

### Ovuliferous reproductive phenology

**Figure 5.**
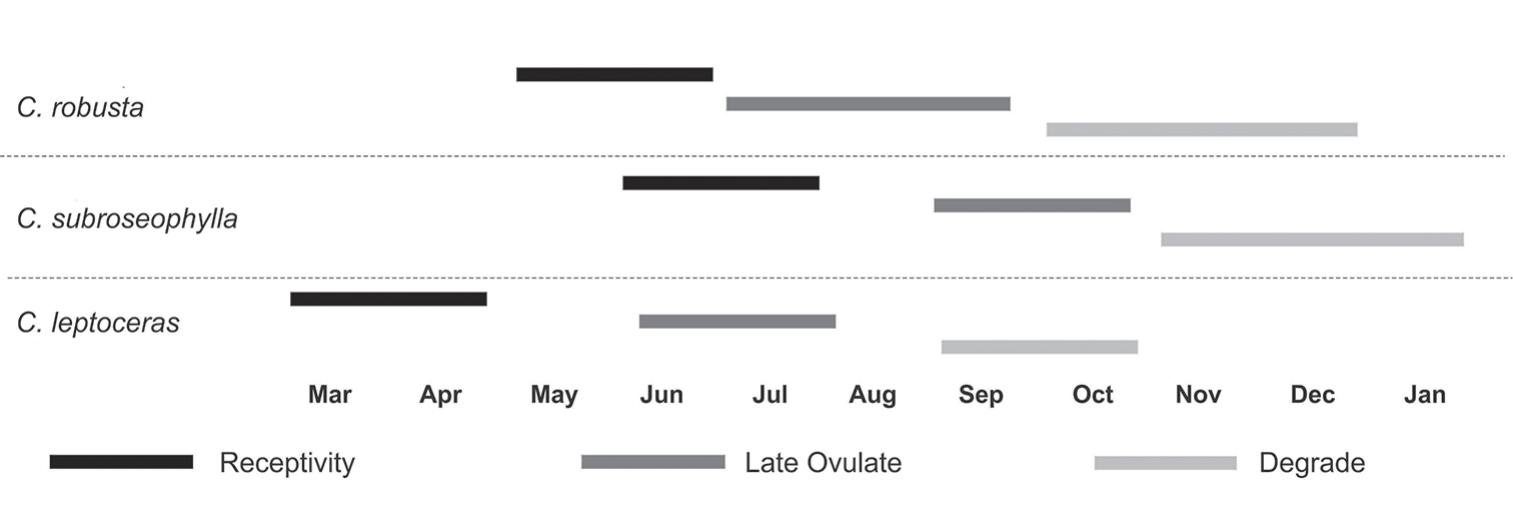
Phenological patterns of ovulate strobili in *Ceratozamia leptoceras* and morphologically similar species.

Overall, the Guerrero taxon has a phenological reproductive pattern that differs from its most morphologically related species (Fig. 5). The phenological cycle of *Ceratozamia robusta* and *C. subroseophylla* is mutually more similar; however, in the first species, the R phase occurs from May to June, whereas in *C. subroseophylla* this phase occurs from June to July. OT and D phases for these species have different offsets (Fig. 5). In contrast, the R phase in the Guerrero taxon occurs from March to April, while the D phase runs from September to October.

### Integrative taxonomic inference of a new *Ceratozamia* species

According to the inferential rules of the ‘taxonomic circle’ in our integrative taxonomy approach, the specimens collected from Guerrero were marked with a ‘red flag’ – i.e. as a hypothetical species demanding test. This hypothesis was corroborated after detection of morphological diagnostic characters, DNA diagnostics and phenological differences, and after the establishment of the particular geographic locality of the collected specimens. Formally, these specimens are recognized here as belonging to a new species based on the presence of (i) distinct morphological qualitative characters, particularly in reproductive structures; (ii) exclusive DNA diagnostic sites in *matK*; (3) distinctions in the phenological pattern in comparison to similar species; and (4) a separate geographical range, which suggests allopatric geographic isolation (i.e. barriers to gene flow). In summary, the new *Ceratozamia* species from Guerrero is diagnosable according to all tested criteria.

## Taxonomic treatment

### Ceratozamia leptoceras

Taxon classificationPlantaeCycadalesZamiaceae

Mart.-Domínguez, Nic-Mor., D.W. Stev. & Lorea-Hern. sp. nov.

D6EC89E2-6ABB-5C73-BB17-C3EE5A0284D8

urn:lsid:ipni.org:names: 77211166-1

Figures 6 , 7 , 8

#### Type.

Mexico. Guerrero: Tlacoachistlahuaca, 3 Km NW of San Pedro Cuitlapan, 1,400 m, 26 Jun. 2019, *L. Martínez-Domínguez & F. Nicolalde-Morejón 1867* ♀ (holotype CIB; isotypes MEXU, NY).

*Ceratozamia leptoceras* is most similar to *C. robusta*, but can be distinguished by its linear membranaceous leaflets and petioles with thin prickles. In addition, *C. leptoceras* is easily distinguished from its congeners by having obconic microsporophylls with a long, linear infertile portion (0.83–0.96 cm), and two thin horns; ovulate strobilus with abundant pubescence at base of megasporophylls, 8–9 orthostichies, and 7–9 sporophylls per orthostichy.

**Figure 6.**
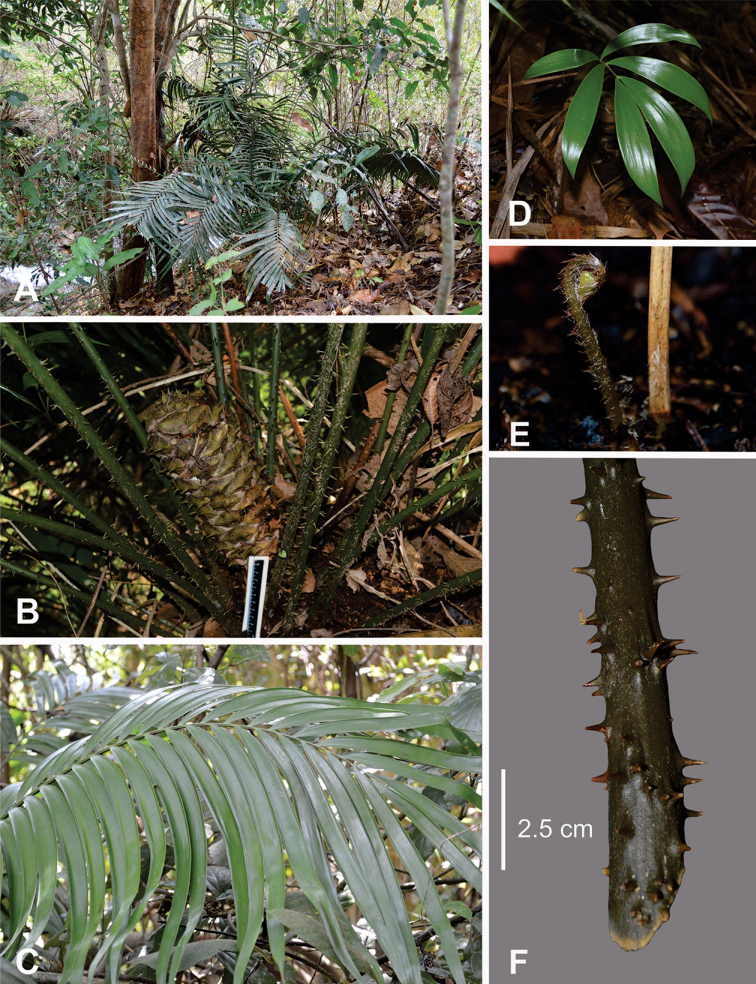
*Ceratozamia leptoceras***A** adult plant in habit **B** ovulate strobilus **C** detail of leaflets **D** seedling **E** ptyxis **F** prickles on petiole.

#### Additional specimens examined

**(paratypes).** Mexico. **Guerrero**: Cochoapa El Grande, 4 km to W-NW of San Pedro by a logging road, 1,170 m, 4 Feb 1984, *F. Lorea-Hernández 2928* (FCME); Tlacoachistlahuaca, San Pedro Cuitlapan, riverbank “Chipili”, 1,200 m, 29 May 2019, *L. Martínez-Domínguez et al. 1756* (CIB), *1757* (CIB, MEXU), *1758* (XAL), *1759* (CIB, MEXU); *F. Nicolalde-Morejón et al. 3173* (XAL), *3174* (FCME), *3175* (CIB); 3 km NW of San Pedro Cuitlapan, 1,400 m, 26 June 2019, *L. Martínez-Domínguez & F. Nicolalde-Morejón 1860*, *1861* (MEXU), *1862–1866* (CIB).

#### Description.

*Stem* epigeous, erect to decumbent, 30–150 cm in length, 11–35 cm in diameter, covered with leaf bases. *Cataphylls* persistent, reddish-brown, densely brownish tomentose abaxially at emergence, pubescent at maturity, triangular, apex acuminate, 9–11 × 2.5–3 cm at base. *Leaves* 7–50, descending, 93.5–281 cm, green at emergence with sparse reddish-brown pubescence, glabrous at maturity. *Petiole* terete, linear, 45–85 cm, armed with long (0.48–0.68 cm) and thin prickles, copperish-green in mature leaves. *Rachis* terete, linear, 75–196 cm, armed with long and thin prickles, green in mature leaves. *Leaflets* 22–61 pairs, linear, abaxially curved, not basally falcate, membranaceous, flat, opposite to subopposite, plane, green, adaxial and abaxial surfaces glabrous, acuminate and symmetric apex, attenuate at base, with conspicuous and green veins; median leaflets 28–43.5 × 1.9–2.8 cm, 1.8–2.8 cm between leaflets; articulations generally copperish-green. *Pollen strobilus* generally solitary (rarely 2), cylindrical, erect, 40–45 cm in length, 6.0–7.8 cm in diameter, brownish-yellow at emergence, yellowish-green with brownish trichomes at maturity; peduncle tomentose, reddish-brown to brown, 13–19 cm in length, 1.5–2.0 cm in diameter; microsporophylls 2.1–2.45 × 1.09–1.30 cm, obconic, non-recurved distal face, fertile portion deeply lobate, infertile portion 0.83–0.96 cm, linear, horns 0.1–0.23 cm, straight, thin, 0.44–0.56 cm between horns, 180–230 sporangia on abaxial side. *Ovulate strobilus* solitary, cylindrical, erect, 23.5–28 cm in length, 9.5–11 cm in diameter, brownish-green with greyish-black trichomes at emergence, copperish-green with greyish-black pubescence at maturity, acute apex; peduncle tomentose, brown, 11–16 cm in length, 1.5–2.0 cm in diameter; megasporophylls 56–81, 8–9 orthostichies (column), 7–9 sporophylls per column, 4.9–5.6 × 2.2–2.6 cm, prominent distal face, horns 0.63–0.81 cm, straight, 0.95–1.35 cm between horns, straight angle between horns. *Seeds* ovoid, 2.43–2.71 cm in length, 1.4–1.8 cm in diameter, sarcotesta whitish-pink when immature, light brown at maturity.

**Figure 7.**
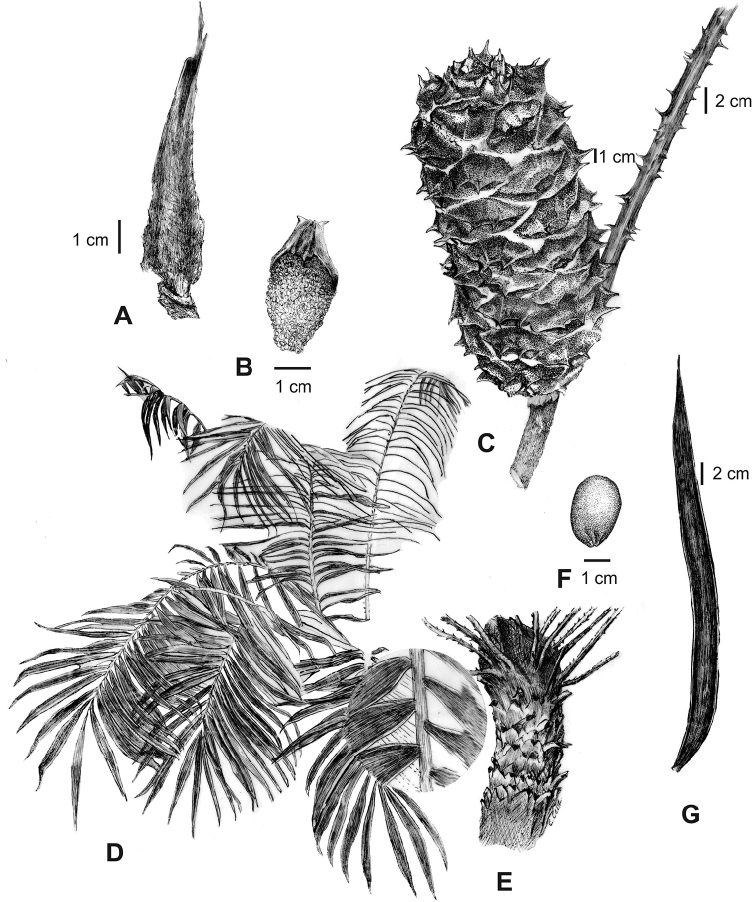
Illustration of *Ceratozamia leptoceras***A** cataphyll **B** microsporophyll **C** ovulate strobilus **D** leaves and detail of leaflets **E** stem **F** seed **G** leaflet. This illustration is based on *L. Martínez-Domínguez & F. Nicolalde-Morejón 1867*, with exception microsporophyll, which is based on *L. Martínez-Domínguez et al. 1757*.

#### Etymology.

The specific epithet alludes to microsporophylls horns shape, which are short and thin. This name comes from the Greek “lepto”, which means thin or fine, and “ceras” in reference to horns.

#### Distribution and habitat.

Only known from Guerrero, Mexico, on the karstic rocks within the elevation range of 1,170–1,400 m of the Sierra Madre del Sur subprovince of the Guerreran district (Morrone 2017) (Fig. 1). This species occurs in cloud forest. The climate type is (A) C (w2)-semi-warm temperate subhumid with summer rains, and the annual precipitation is from 2, 000 to 2, 500 mm (García 2004).

#### Phenology.

The leaves are produced in groups of 9 to 15 and mature almost simultaneously. Ovulate strobili mature from June to July; seeds mature from August to September. Pollen strobili mature from January to May.

#### Common names.

The common local name for this species by the “Mixteco” ethnic group is *Shalukaá*.

**Figure 8.**
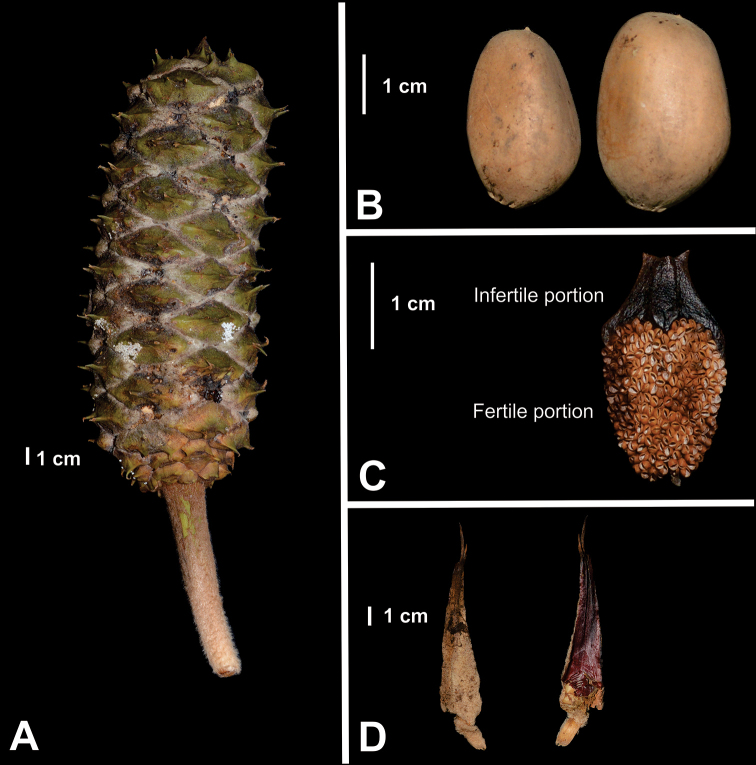
*Ceratozamia leptoceras***A** detail of ovulate strobilus and megasporophylls **B** seeds **C** abaxial view of microsporophylls **D** cataphylls **A, B, D** are based on *L. Martínez-Domínguez & F. Nicolalde-Morejón 1867*; C is based on *L. Martínez-Domínguez et al. 1757*.

### Taxonomic keys to *Ceratozamia leptoceras* and morphologically related species

Key for vegetative plants

**Table:** 

1	Leaflets coriaceous	**2**
–	Leaflets membranaceous to papyraceous	**3**
2	Leaflets keeled; petiole with long (0.3–0.6 cm) prickles robust and abundant (> 50)	***C. brevifrons***
–	Leaflets flat; petiole with short (0.1–0.2 cm) prickles thin and sparse (< 40)	**4**
4	Leaves ascending; new leaves yellowish-green at emergence. Cataphylls pubescent to tomentose	***C. delucana***
–	Leaves descending; new leaves light-green at emergence. Cataphylls pubescent to scarcely pubescent	***C. mexicana***
3	Leaves descending; petiole unarmed to armed with thin prickles	**5**
–	Leaves ascending; petioles armed with robust prickles	**8**
5	Leaflets linear, membranaceous. New leaves green with copperish-green petiole and rachis	***C. leptoceras***
–	Leaflets lanceolate, papyraceous. New leaves green	**6**
6	Petiole unarmed to armed with scarce prickles (< 15)	***C. whitelockiana***
–	Petiole armed with abundant prickles (> 20)	**7**
7	Leaflets with adaxial side glabrous; leaflets in median portion inserted at acute angle along rachis	***C. mixeorum***
–	Leaflets glaucous; leaflets in median portion inserted at right angle along rachis	***C. delucana***
8	New leaves dark brown at emergence. Leaflets in median and apical portion abaxially curved	***C. robusta***
–	New leaves yellowish brown at emergence. Leaflets abaxially curved in median and apical portion mostly planar	***C. subroseophylla***

Key for microsporangiate plants

**Table:** 

1	Microsporophylls discoid, infertile portion orbicular	***C. delucana***
–	Microsporophylls obconic, infertile portion rounded to linear	**2**
2	Microsporophylls with straight horns	**3**
–	Microsporophylls with recurved horns	**4**
4	Pollen strobili 60–70 cm long; microsporophylls with infertile portion deeply lobate	***C. robusta***
–	Pollen strobili < 50 cm long; microsporophylls with infertile portion lobate	**6**
6	Peduncle of pollen strobili > 3.5 cm, tomentose reddish-brown to light brown	**7**
–	Peduncle of pollen strobili <3 cm, glabrous to scarcely pubescent	***C. whitelockiana***
7	Pollen strobili green with black trichomes, peduncle 3.5–5 cm long	***C. mexicana***
–	Pollen strobili light green with brown-reddish trichomes, peduncle 10–15 cm long	***C. subroseophylla***
3	Microsporophylls with infertile portion linear	**5**
–	Microsporophylls with infertile portion rounded	***C. mixeorum***
5	Microsporophylls with infertile portion of 0.4–0.61 cm long, horns 0.24–0.33 cm long	***C. brevifrons***
–	Microsporophylls with infertile portion of 0.83–0.96 cm long, horns 0.1–0.23 cm long	***C. leptoceras***

## Discussion

In this synthesis of *Ceratozamia*, we constructed a database for the genus based with geographic, morphological, and molecular data through the use of comparative and character-based DNA barcoding methods. In addition, we investigated the potential value of phenological reproductive patterns for species delimitation; this type of ecological information has been scarcely studied in cycads (Stevenson 1981; Clugston et al. 2016) or in integrative taxonomy studies of gymnosperms. Considering the different male plants in a population, the phenology of pollen strobili shows a broader cycle at the population level; the lifespan of a pollen strobilus is shorter than for ovulate strobili, and an open pollen phase is prolonged in different individual plants (Martínez-Domínguez et al. 2018b). In contrast, duration of ovulate strobili exhibits high specificity in the receptivity phase, which suggests that these data are taxonomically informative. Our findings on the length of the lifespan of reproductive structures and phenological phases coincide with our previous observations in *C. tenuis* (Martínez-Domínguez et al. 2018b). *Ceratozamia leptoceras* has a different phenological pattern for ovulate strobili compared to its morphologically similar congeners, which perhaps represents a reproductive barrier in the field (Fig. 5). Unfortunately, these data are not available for all *Ceratozamia* species.

Despite the species diversity of *Ceratozamia* and its restricted geographic distribution, a formal infrageneric classification for the genus has not been proposed. This is mainly because previously reconstructed phylogenetic relationships display remarkable differences in the number of clades and the members of the clades (cf. González and Vovides 2002, 2012; Medina-Villarreal et al. 2019). This situation has led to an incorrect identification of specimens that unfortunately has limited the inference of evolutionary relationships, with contradictory results inexplicably unrecognized (cf. Medina-Villarreal and González-Astorga 2016; Medina-Villarreal et al. 2019). The groups within the genus are based on similarities in geographic distribution and morphology (Vovides et al. 2004); however, *Ceratozamia* species complexes defined by these authors are more influenced by geography. In this work, we follow the proposal of Stevenson et al. (1986) to address morphology in this genus. Our morphological description for all *Ceratozamia* species recognized tree groups based on the affinity of morphological characters, both vegetative and reproductive, which facilitates comparisons between species.

Under integrative taxonomy criteria (*sensu* De Salle et al. 2005), we have proposed a new *Ceratozamia* species from the Sierra Madre del Sur. This biogeographic province is a salient area in terms of gymnosperm diversity in the country (Contreras-Medina 2016). This new record for the genus in Guerrero represents an expansion of the distribution pattern for the genus on the Pacific seaboard, which opens new questions on the influence of mountain systems on the diversity of the Mexican flora. Our survey shows that the vast majority of *Ceratozamia* species grow at relatively high elevations (Fig. 2); the genus has standard elevation gradient patterns and does not exhibit occurrences at higher elevations or lower elevations. Therefore, it appears that *Ceratozamia* is poorly adapted at lower and very high elevations (Fig. 2). Species with the lowest elevation distributions occur in southern Mexico and Central America, and those at higher elevations occur in the SMO, Highlands of Chiapas, and SMS. This atypical distribution pattern seems to be favored by mountain regions, mainly in areas with cloud forest.

Historically, taxonomic research in the genus *Ceratozamia* has been characterized by difficulties in species identification (Miquel 1868). Recently, new species have been described (e.g. Martínez-Domínguez et al. 2018a) but only some of them have been evaluated. Particularly, in relation to DNA datasets, only 19 species could be diagnosed with ITS+*matK*. Other loci already tested (*psbK/I*, *rpoC1*, *rbcL* and *atpF/H*) have not provided enough variation for improved resolution (see Nicolalde-Morejón et al. 2011; Martínez-Domínguez et al. 2017). As an approach to molecular species identification, character-based DNA barcoding is susceptible to the addition of new sequences (Nicolalde-Morejón et al. 2010); therefore, further sequencing of new loci (e.g. single-copy nuclear genes; Salas-Leiva et al. 2014) could contribute new molecular diagnostic characters and improved resources for automated species identification in *Ceratozamia* and other cycad genera.

In terms of morphological taxonomic evidence, vegetative characters have been widely used in the genus to identify species (Vovides et al. 2004; Whitelock 2004; Pérez-Farrera et al. 2009). However, these characters are polymorphic in some species, which hinders the construction of dichotomous keys (Martínez-Domínguez et al. 2017). The classification of strictly allopatric taxa, as is the case in some *Ceratozamia* species, will always remain arbitrary to some degree so that the evaluation of new characters is a pressing task. In this work, we introduced previously unreported reproductive characters such as the internal parts of the strobilus, which have not been described before in detail for the genus *Ceratozamia* (Fig. 4; Table 1). Comparisons of pollen strobili and the microsporophylls among different species indicated strong morphological divergence limited to certain groups of related species (Fig. 4). For this reason, we only present a key for the species group that includes the newly described taxon. Thus, it is necessary to explore these reproductive characters for the rest of the species in the genus. Keys based on vegetative features still preserve their relevance due to the dioic condition of these gymnosperms, and the lack of reproductive structures in materials deposited at the consulted herbaria (Morettii et al. 1989). However, we consider that, in view of the complexity of vegetative morphological variation, keys based on reproductive characters could function as excellent support tools in the systematics of cycad genera such as *Ceratozamia*.

Species delimitation in *Ceratozamia* exhibits a high degree of complexity (Whitelock 2004). The morphological and molecular evidence available for the genus at present does not allow the diagnosability of all species. For example, *C. hildae* is a morphologically distinctive species, but lacks molecular diagnostics; in contrast, *C. vovidesii* and *C. mirandae* are species that share many morphological affinities, but variation at the molecular level facilitates their taxonomic differentiation (Table 3). This taxonomic-systematic scenario points to the need to explore new molecular markers and the evaluation of variation between populations with morphological, molecular, and phenological evidence for some closely related species. In particular, there are some populations in Oaxaca and Chiapas that still await closer inspection. In the present work, 31 species have been recognized for the genus (Stevenson et al. 1986; Nicolalde-Morejón et al. 2014; Vovides et al. 2016; Pérez-Farrera et al. 2017; Martínez-Domínguez et al. 2017, 2018a), with *C. becerrae* and *C. microstrobila* treated as synonyms of *C. zoquorum* and *C. latifolia*, respectively (Stevenson et al. 1986; Martínez-Domínguez et al. 2017, 2018a).

## Conclusions and future directions

This study adds to recent research that suggests the significant role of topography in SMS as a speciation driver in shaping its high species diversity (Santiago-Alvarado et al. 2016). The geographical barriers and ecological changes in this biogeographic province could have allowed the intermingling of different species; these processes could have favored the development of a number of centers of endemism (Morrone 2010; Santiago-Alvarado et al. 2016).

Our integrative taxonomic assessment provided support for the recognition of a new species, *Ceratozamia leptoceras*. However, the taxonomic complexity of the genus indicates the need of further systematic revisions using multiple sources of evidence, particularly in some groups of species with problematic boundaries. In addition, we have demonstrated the value of investigating ovulate and pollen strobili – particularly, microsporophylls – for the construction of refined morphological matrices for *Ceratozamia*; and finally, that the construction of dichotomous keys with vegetative characters should consider variation at the population level.

## Supplementary Material

XML Treatment for Ceratozamia leptoceras

## Appendix I

**Table:** Localities of sampled populations for morphologically similar *Ceratozamia* species to Guerrero taxon.

**Species**	**State**	**Municipality**	**Population**	**Elevation (m)**
*C. robusta*	Chiapas	Berriozábal	Cuchumbak	1,129
*C. robusta*	Chiapas	Tuxtla Gutiérrez	Cañón del Sumidero	1,263
*C. robusta**	Chiapas	San Fernando	Cuauhtémoc	1,200
*C. subroseophylla*	Veracruz	Santiago Tuxtla	Hill El Vigía	474
*C. subroseophylla*	Veracruz	Santiago Tuxtla	Sinapan	425
Guerrero taxon	Guerrero	Tlacoachistlahuaca	Near Riverbank “Chipili”	1,200
Guerrero taxon	Guerrero	Tlacoachistlahuaca	Near San Pedro Cuitlapan	1,400

* Population not monitored in field for reproductive phenology data.

## Supplementary materials

Supplementary material 1 File S1. GenBank accession numbers of sequences used in the analyses for ITS and matK, respectively. Sequences were generated by this study are in bold. Data type GenBank accession numbers of sequences used in the analyses https://binary.pensoft.net/file/444870This dataset is made available under the Open Database License (http://opendatacommons.org/licenses/odbl/1.0/). The Open Database License (ODbL) is a license agreement intended to allow users to freely share, modify, and use this Dataset while maintaining this same freedom for others, provided that the original source and author(s) are credited.Lilí Martínez-Dominguez, Fernando Nicolalde-Morejón

Supplementary material 2 Fig. S1. Strict consensus tree of nine equally parsimonious trees Data type Strict consensus tree Explanation note Heuristic searches were conducted with 1000 random-addition, tree bisection-reconnection (TBR) branch swapping, and collapse zero-length branches off. All characters were treated as equally weighted and unordered. https://binary.pensoft.net/file/444871This dataset is made available under the Open Database License (http://opendatacommons.org/licenses/odbl/1.0/). The Open Database License (ODbL) is a license agreement intended to allow users to freely share, modify, and use this Dataset while maintaining this same freedom for others, provided that the original source and author(s) are credited.Lilí Martínez-Dominguez, Fernando Nicolalde-Morejón

Supplementary material 3 File S2 Explanation note Herbarium specimens consulted for phenology data. https://binary.pensoft.net/file/444889This dataset is made available under the Open Database License (http://opendatacommons.org/licenses/odbl/1.0/). The Open Database License (ODbL) is a license agreement intended to allow users to freely share, modify, and use this Dataset while maintaining this same freedom for others, provided that the original source and author(s) are credited.Lilí Martínez-Dominguez, Fernando Nicolalde-Morejón

Supplementary material 4 Table S1. Data type DNA diagnostic sites https://binary.pensoft.net/file/444873This dataset is made available under the Open Database License (http://opendatacommons.org/licenses/odbl/1.0/). The Open Database License (ODbL) is a license agreement intended to allow users to freely share, modify, and use this Dataset while maintaining this same freedom for others, provided that the original source and author(s) are credited.Lilí Martínez-Dominguez, Fernando Nicolalde-Morejón
