# Supplementary material for: A novelty in Ceratozamia (Zamiaceae, Cycadales) from the Sierra Madre del Sur, Mexico: biogeographic and morphological patterns, DNA barcoding and phenology
Source: PhytoKeys. 2020 Aug 21;156:1–25. doi: 10.3897/phytokeys.156.53502 (PMC7471474; doi:10.3897/phytokeys.156.53502)
Supplement: Supplementary material 1 — File S1. GenBank accession numbers of sequences used in the analyses for ITS and matK, respectively. Sequences were generated by this study are in bold. [file phytokeys-156-001-s001.docx]

**Supplementary material 1.** GenBank accession numbers of sequences used in the analyses for *ITS* and *matk*, respectively. Sequences were generated by this study are in bold.

*Ceratozamia alvarezii* GU807372, GU807321;

*C. brevifrons* KR611523,

***C. chamberlainii*** ***

*C. chimalapensis* GU807374, GU807323;

*C. decumbens* GU807375, GU807324;

***C. delucana*** *

*C. euryphyllidia* GU807376, GU807325;

*C. fuscoviridis* KR611524,

*C. hildae* GU807377, GU807326;

*C. hondurensis* MN047246,

*C. huastecorum* GU807378, GU807327;

*C. kuesteriana* GU807379, GU807328;

*C. latifolia* GU807380, GU807329;

*C. matudae* GU807381, GU807330;

***C. mexicana*** *

*C. miqueliana* GU807384, GU807333;

*C. mirandae* GU807385, GU807334;

*C. mixeorum* GU807386, GU807335;

***C.* *leptoceras*** ***

*C. morettii* GU807387, GU807336;

*C. norstogii* GU807388, GU807337;

*C. robusta* GU807389, GU807338;

*C. sabatoi* GU807390, GU807339;

*C. santillanii* KR611525,

*C. subroseophylla* KR611527,

*C. tenuis* GU807382, GU807331,

***C. totonacorum*** *

*C. vovidesii* GU807391, GU807340;

*C. whitelockiana* KR611526, GU807341;

*C. zaragozae* GU807392, GU807342;

*C. zoquorum* GU807393, GU807343.
