# Supplementary figures and images for: A novelty in Ceratozamia (Zamiaceae, Cycadales) from the Sierra Madre del Sur, Mexico: biogeographic and morphological patterns, DNA barcoding and phenology
Source: PhytoKeys. 2020 Aug 21;156:1–25. doi: 10.3897/phytokeys.156.53502 (PMC7471474; doi:10.3897/phytokeys.156.53502)

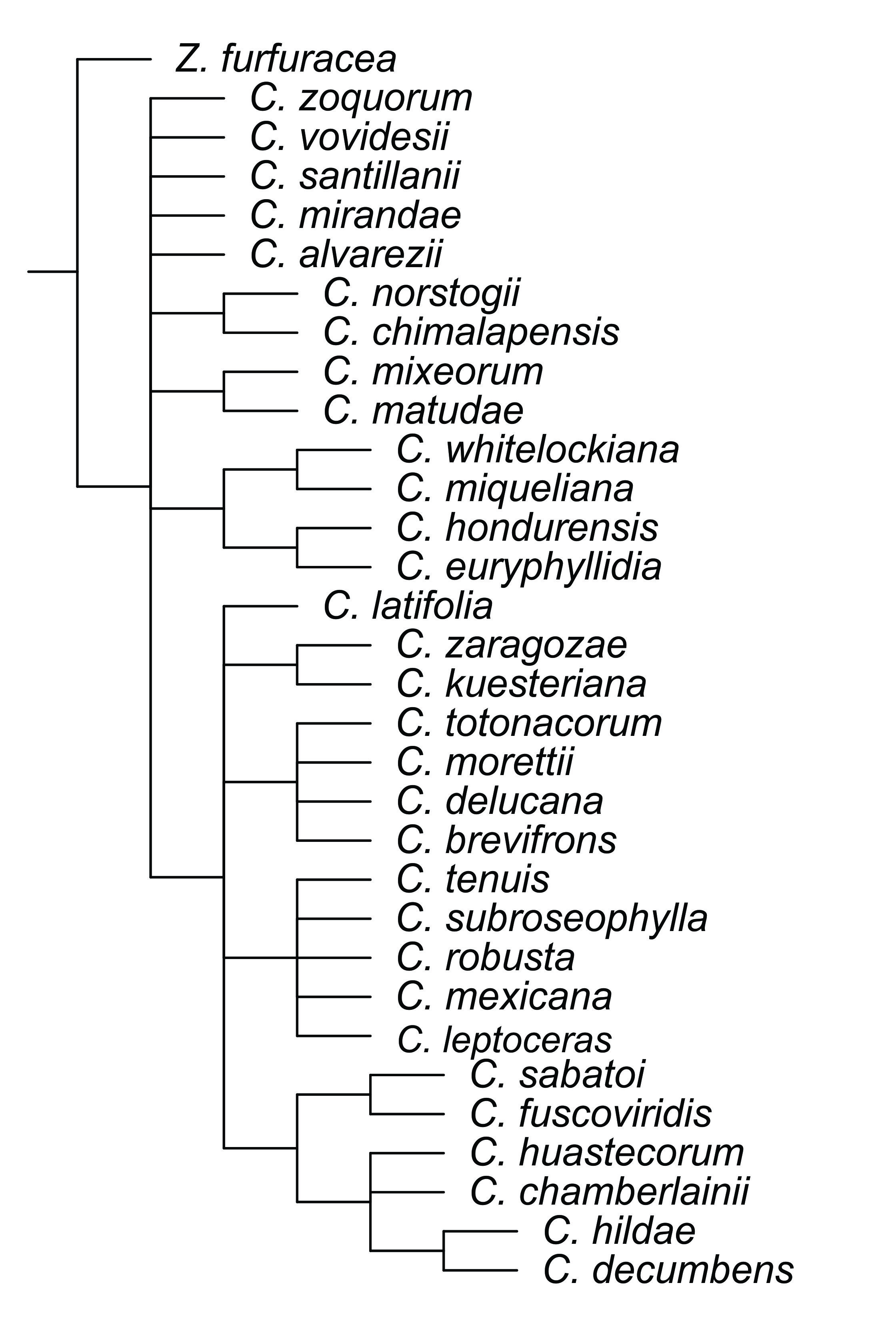

Supplement: Supplementary material 2 — Fig. S1. Strict consensus tree of nine equally parsimonious trees [file phytokeys-156-001-s002.jpg]
