# Supplementary material for: A novelty in Ceratozamia (Zamiaceae, Cycadales) from the Sierra Madre del Sur, Mexico: biogeographic and morphological patterns, DNA barcoding and phenology
Source: PhytoKeys. 2020 Aug 21;156:1–25. doi: 10.3897/phytokeys.156.53502 (PMC7471474; doi:10.3897/phytokeys.156.53502)
Supplement: Supplementary material 3 — File S2 [file phytokeys-156-001-s003.docx]

**Supplementary Material 3.** Herbarium specimens consulted for phenology data.

*C. robusta: M. A. Pérez-Farrera 30,* (CH, MEXU, XAL), *293* (MEXU), *1266a* (XAL); *J. García F. 720* (CIB, XAL); *René Alberto Palestina & I. Acosta 2707* (XAL); *Brigada T. Walters s/n* (XAL), *E. Martínez S. 12067* (MEXU), *A. P. Vovides & J. I. Calzada 481* (XAL).

*C. subroseophylla*: *M. Sousa 3645* (MEXU), *Helia Bravo 26* (MEXU), *T. W. Walters et al., TW-2001-17* (MEXU, XAL), *M. Vázquez-Torres 3579, 4122* (CIB), *M. A. García B., et al. 813* (XAL), *César I. Carvajal & Mauricio Juárez F. 796* (CIB), *J. F. Ortega O. et al., 358* (XAL).
